# Tumefactive multiple sclerosis requiring emergent biopsy and histological investigation to confirm the diagnosis: a case report

**DOI:** 10.1186/1752-1947-6-104

**Published:** 2012-04-06

**Authors:** So Yamada, Shoko Merrit Yamada, Hiroshi Nakaguchi, Mineko Murakami, Katsumi Hoya, Akira Matsuno, Kazuto Yamazaki, Yasuo Ishida

**Affiliations:** 1 Department of Neurosurgery, Teikyo University Chiba Medical Center, 3426-3 Anesaki, Ichihara-city, Chiba-prefecture 299-0111, Japan; 2 Department of Pathology, Teikyo University Chiba Medical Center, 3426-3 Anesaki, Ichihara-city, Chiba-prefecture 299-0111, Japan

## Abstract

**Introduction:**

Tumefactive multiple sclerosis is a demyelinating disease that demonstrates tumor-like features on magnetic resonance imaging. Although diagnostic challenges without biopsy have been tried by employing radiological studies and cerebrospinal fluid examinations, histological investigation is still necessary for certain diagnosis in some complicated cases.

**Case presentation:**

A 37-year-old Asian man complaining of mild left leg motor weakness visited our clinic. Magnetic resonance imaging demonstrated high-signal lesions in bilateral occipital forceps majors, the left caudate head, and the left semicentral ovale on fluid-attenuated inversion recovery and T2-weighted imaging, and these lesions were enhanced by gadolinium-dimeglumin. Tumefactive multiple sclerosis was suspected because the enhancement indistinctly extended along the corpus callosum on magnetic resonance imaging and scintigraphy showed a low malignancy of the lesions. But oligoclonal bands were not detected in cerebrospinal fluid. In a few days, his symptoms fulminantly deteriorated with mental confusion and left hemiparesis, and steroid pulse therapy was performed. In spite of the treatment, follow-up magnetic resonance imaging showed enlargement of the lesions. Therefore, emergent biopsy was performed and finally led to the diagnosis of demyelinating disease. The enhanced lesion on magnetic resonance imaging disappeared after one month of prednisolone treatment, but mild disorientation and left hemiparesis remained as sequelae.

**Conclusions:**

Fulminant aggravation of the disease can cause irreversible neurological deficits. Thus, an early decision to perform a biopsy is necessary for exact diagnosis and appropriate treatment if radiological studies and cerebrospinal fluid examinations cannot rule out the possibility of brain tumors.

## Introduction

Tumefactive multiple sclerosis (tMS) is a demyelinating disease. Because of its tumor-like features on magnetic resonance imaging (MRI) [1,2], histological investigation had played an important role for definite diagnosis of tMS [3,4]. Recently, without biopsy, some tMS cases were diagnosed by magnetic resonance spectroscopy (MRS), positron emission tomography (PET), cerebrospinal fluid (CSF) examination, and response to steroid treatment [5-9]. However, the diagnosis of tMS without histological confirmation is no more than speculation in some complicated cases. We report the case of a patient who had tMS and who required emergent biopsy for exact diagnosis because of rapid enlargement of lesions on MRI after steroid pulse therapy.

## Case presentation

**Figure 1:**
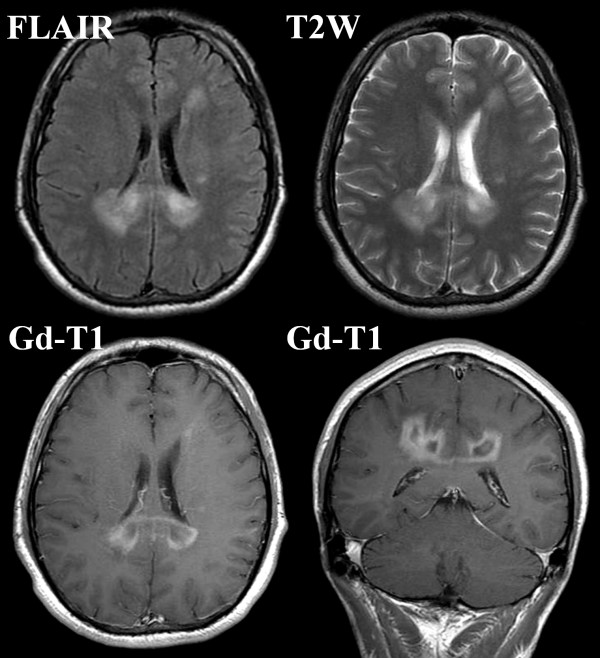
**Pre-operative magnetic resonance imaging**. High-intensity areas in bilateral occipital forceps majors, the left caudate head, and the left semicentral ovale are shown in both fluid-attenuated inversion recovery (FLAIR) and T2-weighted (T2W) imaging. These lesions are enhanced indistinctly by gadolinium-dimeglumin (Gd).

**Figure 2:**
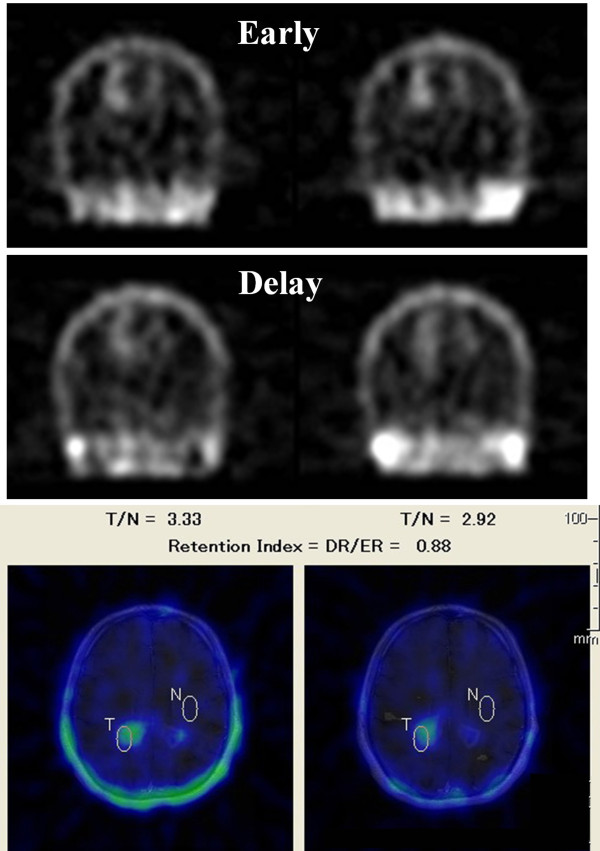
**Uptake of thallium in the lesions on ^201^Tl scintigraphy**. Definite uptake of thallium is identified in the lesions. However, the retention index (ratio of thallium uptake between late and early phases) is 0.88, suggesting a low possibility of malignancy.

A 37-year-old Asian man who complained of a two-month history of dizziness visited our clinic. Neurological examinations using the manual muscle test revealed a left leg motor weakness of 5-/5 on the Medical Research Council (MRC) scale [10,11]. MRI demonstrated high-signal lesions in bilateral occipital forceps majors, the left caudate head, and the left semicentral ovale on both fluid-attenuated inversion recovery (FLAIR) and T2-weighted (T2W) imaging (Figure 1, upper frames). These lesions were enhanced by gadolinium-dimeglumin with expansion along the corpus callosum (Figure 1, lower frame). But these enhancements were not as strong as those recognized in malignant brain tumors. Definite uptake of thallium was identified by ^201^Tl scintigraphy in both early and delay phases. However, the retention index (ratio of thallium uptake between delay and early phases) was 0.88, which was too low for malignant brain tumors to be suspected (Figure 2). tMS was suspected on the basis of the observations of MRI and scintigraphy. However, oligoclonal bands were not detected by CSF examinations. For further investigation, MRS and PET were planned for our patient.

**Figure 3:**
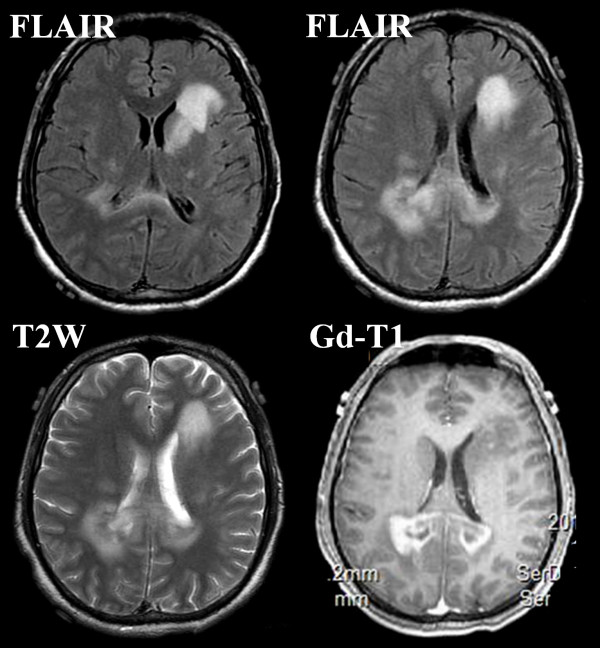
**Magnetic resonance imaging after steroid pulse therapy**. High-intensity areas in the left caudate head, bilateral semicentral ovales, and bilateral occipital forceps majors are clearly enlarged on FLAIR and T2W imaging. Gd-enhanced lesions in the occipital forceps majors are clearly expanded.

A few days later, he was admitted to our hospital because of progression of his left leg motor weakness (4+/5 on the MRC scale). The day after admission, he exhibited mental confusion and left hemiparesis (2/5 on the MRC scale), and three-day consecutive steroid pulse therapy (1000 mg of methylprednisolone sodium succinate and 8 mg of dexamethasone per day) was applied. Despite the treatment, his symptoms did not improve, and MRI showed further enlargement of the lesions (Figure 3). Therefore, without MRS and PET studies, an emergent open biopsy under a navigation system was performed for definite diagnosis.

**Figure 4:**
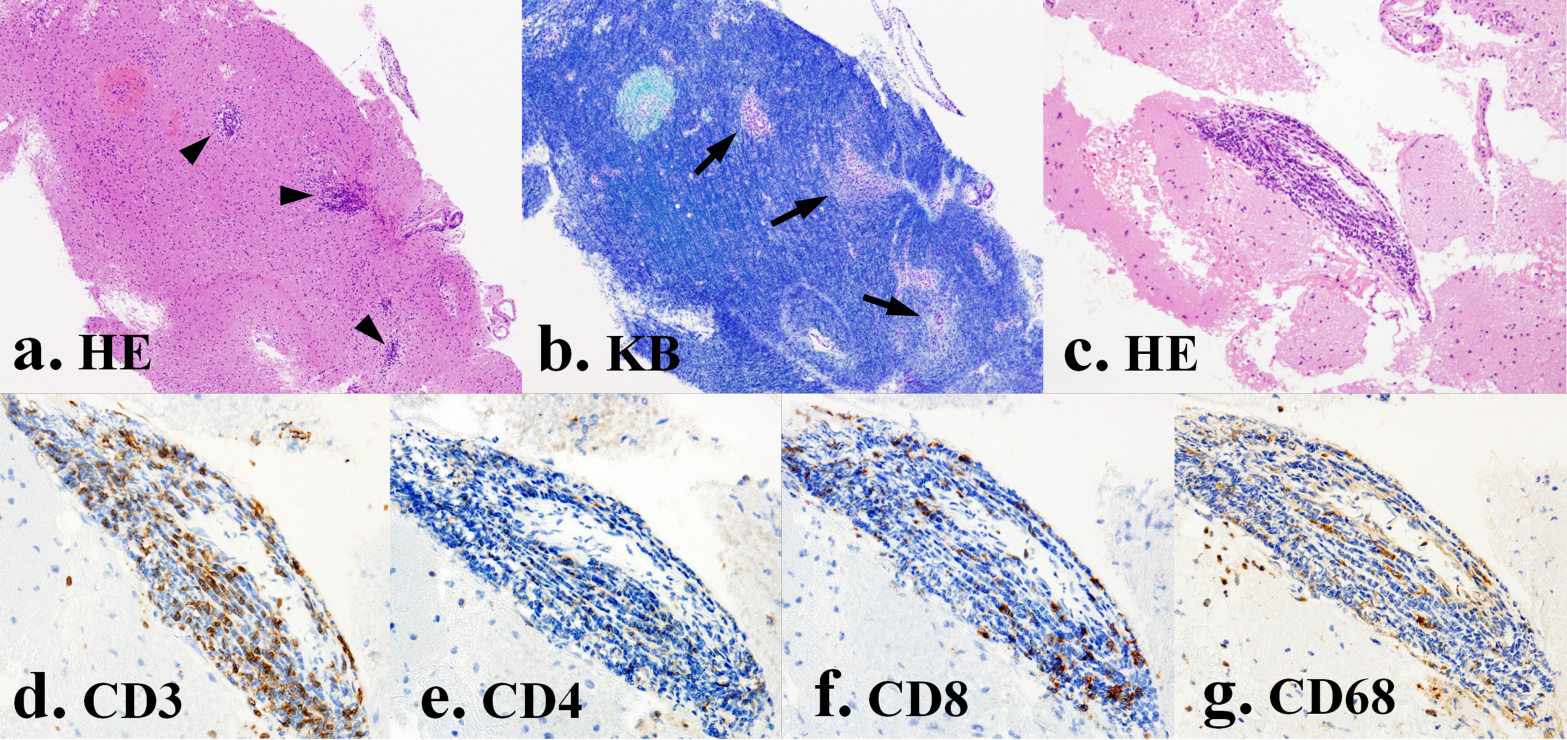
**Definite diagnosis of tMS by histological investigations**. Histology shows proliferation of lymphocytes (arrowheads) without atypical features by hematoxylin-eosin (HE) staining (a). Kluver-Barrera staining demonstrates sporadic defects of myelin (arrows), indicating demyelinations (b). Perivascular lymphocyte proliferation is recognized by HE staining (c). These lymphocytes are composed mainly of CD3-positive T-cell lymphocytes (d), and a mixture of CD4-positive (e) and CD8-positive (f) T-cell lymphocytes rules out monoclonal increase of the lymphocytes. The appearance of macrophages (CD68) in the lesion strongly suggests inflammatory disease (g) rather than malignant lymphoma. CD: cluster of differentiation.

**Figure 5:**
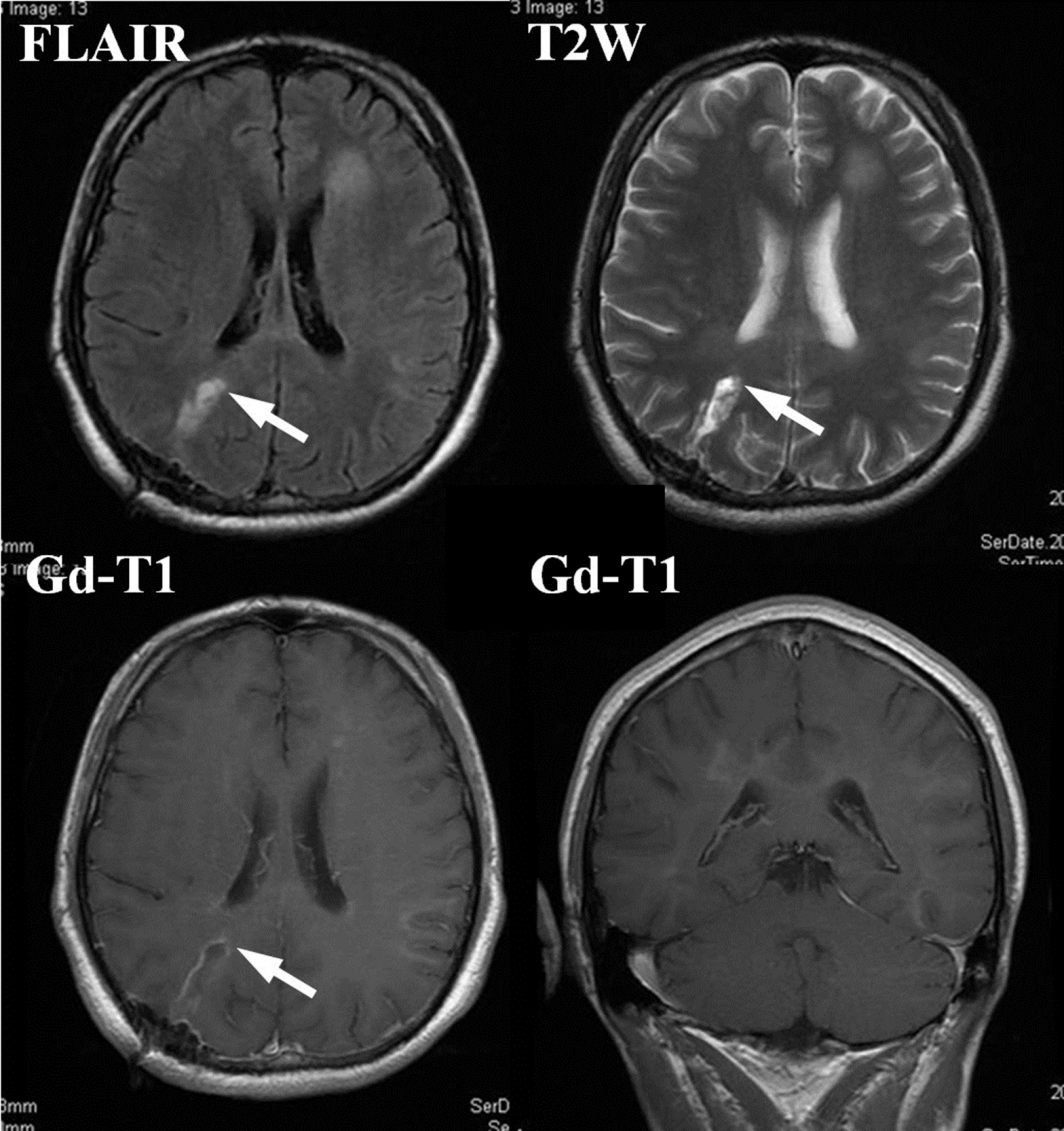
**MRI after one month of steroid therapy**. High-intensity areas in (FLAIR) and T2W imaging considerably shrink and Gd-enhanced lesions completely disappear after one month of steroid treatment. Arrows show the point of biopsy.

Histological investigation showed perivascular proliferation of lymphocytes without atypical features, and Kluver-Barrera staining displayed sporadic absence of the myelin sheath, suggesting demyelinating disease (Figure 4a-c). The majority of the proliferating lymphocytes were T-cell type (Figure 4d), and the existence of lymphocytes positive for both cluster of differentiation (CD) 4 and CD8 (Figures 4e and 4f) ruled out monoclonal increase of the lymphocytes. Furthermore, the presence of macrophages (CD68) strongly implied inflammatory changes in the lesion (Figure 4g). After one month of prednisolone treatment, MRI revealed remarkable shrinking of the lesions (Figure 5), but mild disorientation and left hemiparesis (4+/5 on the MRC scale) remained as sequelae.

## Discussion

The term 'tumefactive MS' is used when the clinical presentation and MRI findings are indistinguishable from those of a brain tumor [12]. Recently, some successful challenges to diagnose tMS by using MRS and PET, without biopsy, have been reported [5-7], and careful follow-up by serial MRI with or without steroid treatment is usually sufficient to establish the diagnosis. In general, tMS lesions respond well to steroids and no radiological evidence of new lesions is identified after the treatment in most patients [13]. Although the clinical course of tMS is various with acute onset, the prognosis of tMS usually does not depend on the clinical presentation [3]. However, in some atypical cases, the radiological diagnosis is no more than speculative if histological diagnosis is not obtained. Butteriss and colleagues [14] reported an interesting case of oligodendroglioma in MS that was diagnosed by surgical removal of the lesion but that had been considered to be tMS on pre-operative MRI. In our case, fulminant deterioration of the clinical symptoms and rapid enlargement of the lesions on MRI in spite of steroid pulse therapy confounded the diagnosis of tMS. When monoclonal bands are not detected in CSF and radiological examinations cannot completely rule out a malignant brain tumor, an early decision to perform a biopsy is required. Once fulminant deterioration of clinical signs and symptoms occurs in a patient with tMS, the neurological deficits can be irreversible.

## Conclusions

To diagnose tMS without histological investigation is an initial approach to the disease. However, early biopsy should not be delayed if radiological examination failed to confirm the diagnosis.

## Abbreviations

CD: cluster of differentiation; CSF: cerebrospinal fluid; MRC: Medical Research Council; MRS: magnetic resonance spectroscopy; MRI: magnetic resonance imaging; PET: positron emission tomography; tMS: tumefactive multiple sclerosis.

## Consent

Written informed consent was obtained from the patient for publication of this manuscript and accompanying images. A copy of the written consent is available for review by the Editor-in-Chief of this journal.

## Competing interests

The authors declare that they have no competing interests.

## Authors' contributions

SY was a major contributor in writing the manuscript. SMY performed surgery, analyzed the data, and revised the manuscript. HN and MM managed the patient, planned imaging studies, and analyzed the data. KH contributed to the conception and interpretation of the disease on the basis of several articles. AM revised the manuscript and provided important intellectual content. KY and YI performed the histological examination and diagnosed the demyelinating disease. All authors read and approved the final manuscript.
